# Supplementary material for: HBV preS Mutations Promote Hepatocarcinogenesis by Inducing Endoplasmic Reticulum Stress and Upregulating Inflammatory Signaling
Source: Cancers (Basel). 2022 Jul 4;14(13):3274. doi: 10.3390/cancers14133274 (PMC9265300; doi:10.3390/cancers14133274)
Supplement: Supplementary file 1 [file cancers-14-03274-s001.zip › cancers-1739074-supplementary.pdf]

Supplementary material

HBV preS mutations promote hepatocarcinogenesis by inducing endoplasmic reticulum stress and up-regulating inflammatory signaling

Supplementary methods

Extraction of viral DNA

Five ml fasting blood from all participants was collected with a vacuum blood collection tube without anticoagulant. The serum was separated by centrifugation at 4°C. HBV DNA was extracted from 200 ml sera by using the serum viral DNA purification kit (PG Biotechnology, Shenzhen, China).

HBV genotyping

HBV genotypes were determined by a multiplex PCR assay that was developed in our laboratory 2. Nested multiplex PCR was conducted for the genotyping of samples with low HBV DNA level. The primers for the first round of nest PCR are 5'-TTTGCGGGTCACCATATTCTTGG-3' and 5'-CGAACCACTGAACAAATGG CACTAG-3', which amplify a fragment of 1106bp from the preS/S region (nt.2815 to nt.705). The product of the first round PCR was used as the template for the multiplex PCR. The amplification conditions of the first round PCR and multiplex PCR were

the same: 94°C for 5 min; 35 cycles which involves denaturing at 94°C for 30s, annealing at 58°C for 60s, and extending at 72°C for 1 min; a terminal extension step at 72°C for 5 min. The products were examined as previously examined.[1]

HBV DNA sequencing and mutation analysis

The HBV preS region gene (nt.1374-nt.1838) was amplified by nested PCR. The primers are listed in Table S1. PCR was performed in 25μl mixture containing 3μl diluted HBV DNA, 1×PCR buffer, 1.5U Taq polymerase (TaKaRa Biotechnology, Dalian, China), 0.2μM each primer (synthesized by Sangon Biotechnology, Shanghai, China), and 0.2mM dNTP (TaKaRa). The amplification was performed with annealing temperature of 58°C for 60s for 35 cycles by using an Autorisierter thermocycler (Eppendorf AG, Hamburg, Germany). DNA was gel purified and sequenced by Sanger sequencing (Maipu, Shanghai, China). Sequence alignment and analysis were performed by using MEGA 4.0 software. The sequence of the wild-type HBV preS1/preS2/S was identified by our previous study. [2]

Construction of recombinant lentivirus expressing wild-type preS1/preS2/S and preS1/preS2/S mutants

The sequences of the wild-type preS1/preS2/S and three preS1/preS2/S mutants were synthesized by Obio Technology (Shanghai, China) and were coloned into the plteni-CMV-GFP-puro lentiviral vector by GeneArt Seamless Cloning and Assembly Kit (Thermo Fisher Scientific, MA). Lentiviruses were packaged using Lentiviral

Packaging System (Invitrogen, Carlsbad, CA) according to the manufacture's instruction. Briefly, the constructed lentiviral vectors were transfected into HEK293T cells together with the MD2G packaging plasmid and PAX2 envelope plasmid by using Lipofectamine 3000 kit (Invitrogen). After incubation for 48h, lentivirus-containing supernatants were harvested and lentiviral particles were concentrated using Lenti-X (Clontech, CA).

Cell culture and the stable transfection of preS1/preS2/S variants

Huh7 and HepG2 cell lines were purchased from the Chinese Academy of Sciences (Shanghai, China). All cells were cultured in Dulbecco's modified Eagle's medium (DMEM, HyClone, Logan, UT) with 10% fetal bovine serum (Gibco, New York, NY) and 1% penicillin/streptomycin (Bio-light, Shanghai, China). Cells were infected with the overexpression lentivirus and the negative control lentivirus for 24h. The cell stains with the stable expression of HBV S protein were screened by 2μg/mL puromycin (Thermo Fisher, Waltham, MA).

Cell proliferation, migration, invasion, and cell cycle assay

Cell proliferation was assessed by the Cell Counting Kit-8 (CCK8) kit (Dojindo, Osaka, Japan). Cells were seeded into the 96-well plates (Corning Incorporated Coster, Kennebunk, ME). Then, 100uL of 10% CCK8-DMEM solution was added and the cells were incubated for 1.5 h. The number of cells was estimated by measuring optical density (OD450) for every 24 h. For colony formation assay, 500 cells were

seeded into 6-well plates and were incubated for 21 days. Cell clones were stained with crystal violet and counted manually. The experiments were performed in triplicate.

The migration and invasion were measured using Transwell inserts (Corning, New York, NY). For the migration assay, a total of 1×10^4 cells were placed in upper wells with 400μL serum-free DMEM. For the invasion assay, the upper chambers were coated with 20% Matrigel (BD Medical Technology, Franklin Lakes, New Jersey) before cell seeding. The lower chamber was filled with 500 μL DMEM (10% FBS). After incubation for 48h, the migrated cells were digested and seeded into 96-wells plates. After incubation for 6h, 100μL DMEM containing 20% MTS (Promega, Madison, WI) and PMS (Promega, Madison, WI) was added. After 2h incubation, absorbance at 490 nm was measured. Five fields were randomly selected and photographed with a microscope at 10 × magnification. Each assay was performed in triplicate.

The cell cycle assay was performed using flow cytometry (Merck Millipore, Rockville, MD). Cells were digested and fixed at -20ºC in 70% ethanol overnight. The cell cycle distribution was measured by flow cytometry using the PI/RNase staining buffer (BD, San Diego, CA) and was analyzed by ModFit LT software (Verity Software House, Topsham, ME). Each assay was performed in triplicate.

Cell apoptosis assay

Cells were stained with V-APC and 7AAD (BD Biosciences, CA, USA) according to

manufacturer's instructions. Flow cytometry measurements were compensated and gated using CytExpert Software (version 2.0, Beckman Coulter). Gating and voltage were carefully set to exclude cell clumps and debris from the analysis with the unstained cells. Compensation was carried out using the single-stained cells for each antibody. The comparison between the fluorescence of single-stained cells and that of the unstained cells was used to determine the position of phenotype and analysis gates. For the analysis of preS1/preS2/S-expressing cells, 10,000 cells were analyzed using the PerCP and ECD channels. Data were plotted as a percentage of $APC^+/PerCP^-$ cells, $APC^-/PerCP^+$ cells, and $APC^+/PerCP^+$ cells.

Real-time quantitative reverse transcription (qRT-PCR) and Western blot

Total RNA was extracted, reversely transcribed into cDNA, and used as the template for qRT-PCR as previously described.[3] Gene expression was relatively quantified using glyceraldehyde-3-phosphate dehydrogenase (*GAPDH*) as an internal control. The primers for qRT-PCR are listed in Table S1. Protein was extracted, quantified, and subjected to Western blot as previously described.[3] The primary antibodies were listed in Table S3. GAPDH was used as the loading control. ImageJ software (version 1.51, https://imagej.nih.gov/ij/) was applied to quantify the signal strength of each band. All experiments were performed in triplicates. The raw data of western blot were presented in Figure S4.

Luciferase reporter assay

Promoter region was defined as 2000 bp upstream of the transcription start site of a given gene based on the Ensembl database (GRCh38.p13, https://asia.ensembl.org/Homo_sapiens). The promoter fragment of STAT3 was synthesized and sequenced by Obio Technology (Shanghai, China). The correct sequences were inserted into the firefly luciferase reporter vector (pGL4.10-basic vector, Promega, Madison, WI) to evaluate the promoter activity. Huh7 and HepG2 cells were co-transfected with the constructed reporter vectors and Renilla luciferase vectors (pRL-TK, Promega) by using Lipofectamine 3000 kit (Invitrogen, Carlsbad, CA). Luciferase activities were tested using a dual-luciferase reporter assay system (Promega). Firefly luciferase activity was normalized to Renilla luciferase activity.

Immunohistochemistry (IHC)

Tissue samples were 10% formalin-fixed and paraffin-embedded for hematoxylin-eosin (H&E) and immunohistochemistry analyses as previously described.[4] Antibodies used were detailed in Table S3. The H&E slides were reviewed by a pathologist (Jianfeng Wu). IHC scores were independently assessed by three investigators who were blind to the experimental data. The staining results were assessed by semi-quantitative methods including staining intensity (0, negative; 1, low; 2, moderate; 3, strong) and the score for the proportion of stained area (0, <10%; 1, 10%-30%; 2, 31%-60%; 3, >60%). The intensity and area of IHC staining were assessed in 5 visual fields. The final IHC score was calculated with the formula:

(staining intensity scores in 5 visual fields + staining area scores in 5 visual fields)/5. Disagreements were resolved by consensus.

HBV capture sequencing

Six tissues of the M2-injected *SB* mice were subjected to HBV-capture sequencing, including three liver tissues the tumor-free mice and three tumor tissues from the mice developing tumor.. DNA was extracted from tissues using Genomic DNA Mini Kit (Invitrogen, Carlsbad, CA). The DNA libraries were constructed using the Fast Library Prep Kit (iGeneTech, Beijing, China). The capture probes of HBV panel were synthesized by iGeneTech. The libraries were captured using the probes of HBV panel and TargetSeq one enrichment kits (iGeneTech). The HiSeqTM 2500 platform (Illumina, San Diego, CA) was applied for the sequencing. The breakpoints of HBV integration were detected using VERSE software.[5] The annotation for the integrated breakpoints was performed with ANNOVAR software.[6]

Gene expression profiling analysis

Mouse genes were converted to human homologs using the HUGO Gene Nomenclature Committee database. For both mouse microarray data and human cell microarray data, differently expressed genes between the WT group and each of the mutant preS1/preS2/S groups were identified using the edgeR package.[7] Among the differentially expressed genes identified in *SB* mice, five oncogenes were selected for qRT-PCR validation, including *Wnt family member 4 (WNT4), ETS homologous*

factor (EHF), phosphoglycerate dehydrogenase (PHGDH), laminin subunit gamma 2 (LAMC2), and mucin 13 (MUC13).[8-12] Gene Ontology (GO) analysis was performed using Panther GO (http://geneontology.org/page/go-enrichment-analysis). Kyoto Encyclopedia of Genes and Genomes (KEGG) pathway analysis was performed utilizing the KEGG pathway database (http://www.genome.jp/kegg/).

Supplementory figure legends

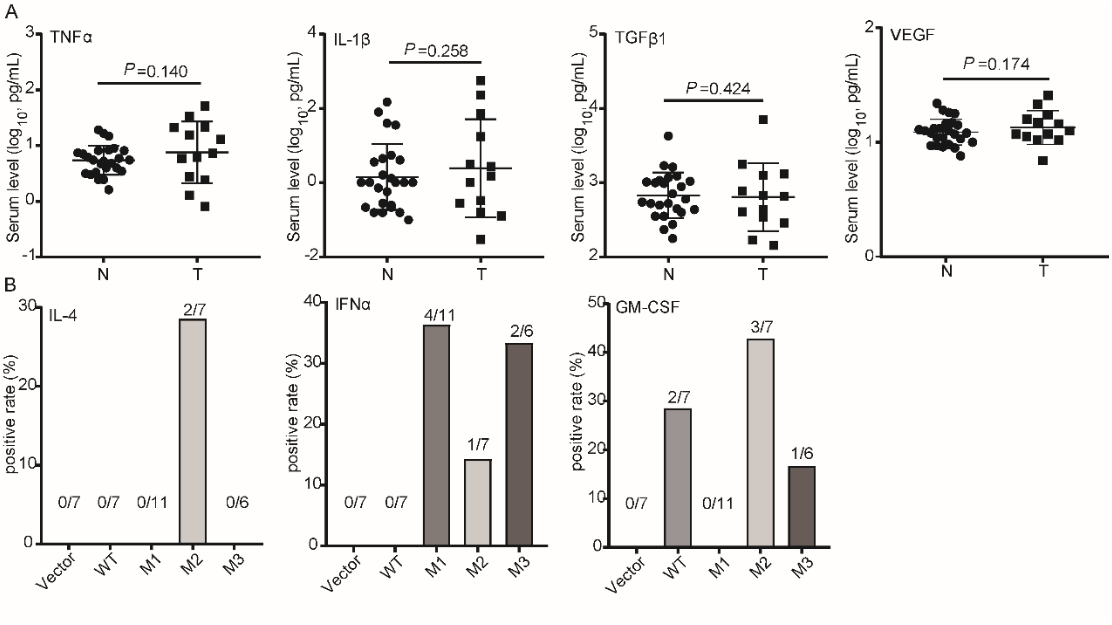

Figure S1. The construction of *Sleeping Beauty* vector.

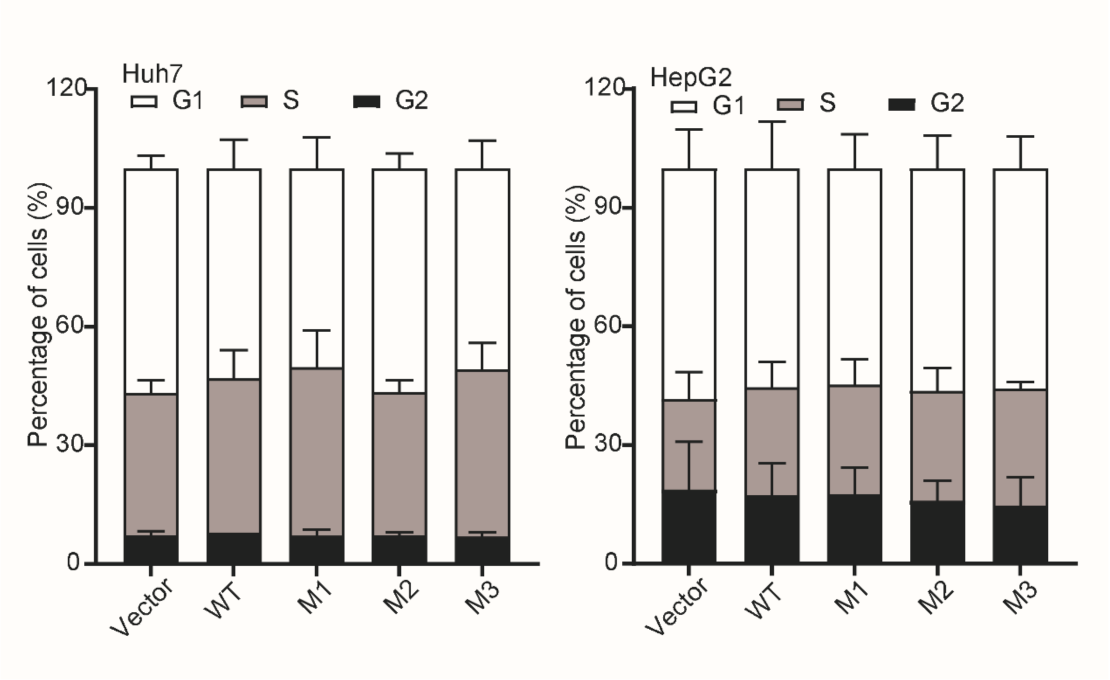

Figure S2. Effect of preS2 deletion on HCC occurrence in patients with antiviral treatment. (A) In patients with INFα treatment. (B) In patients with long-term NAs treatment.

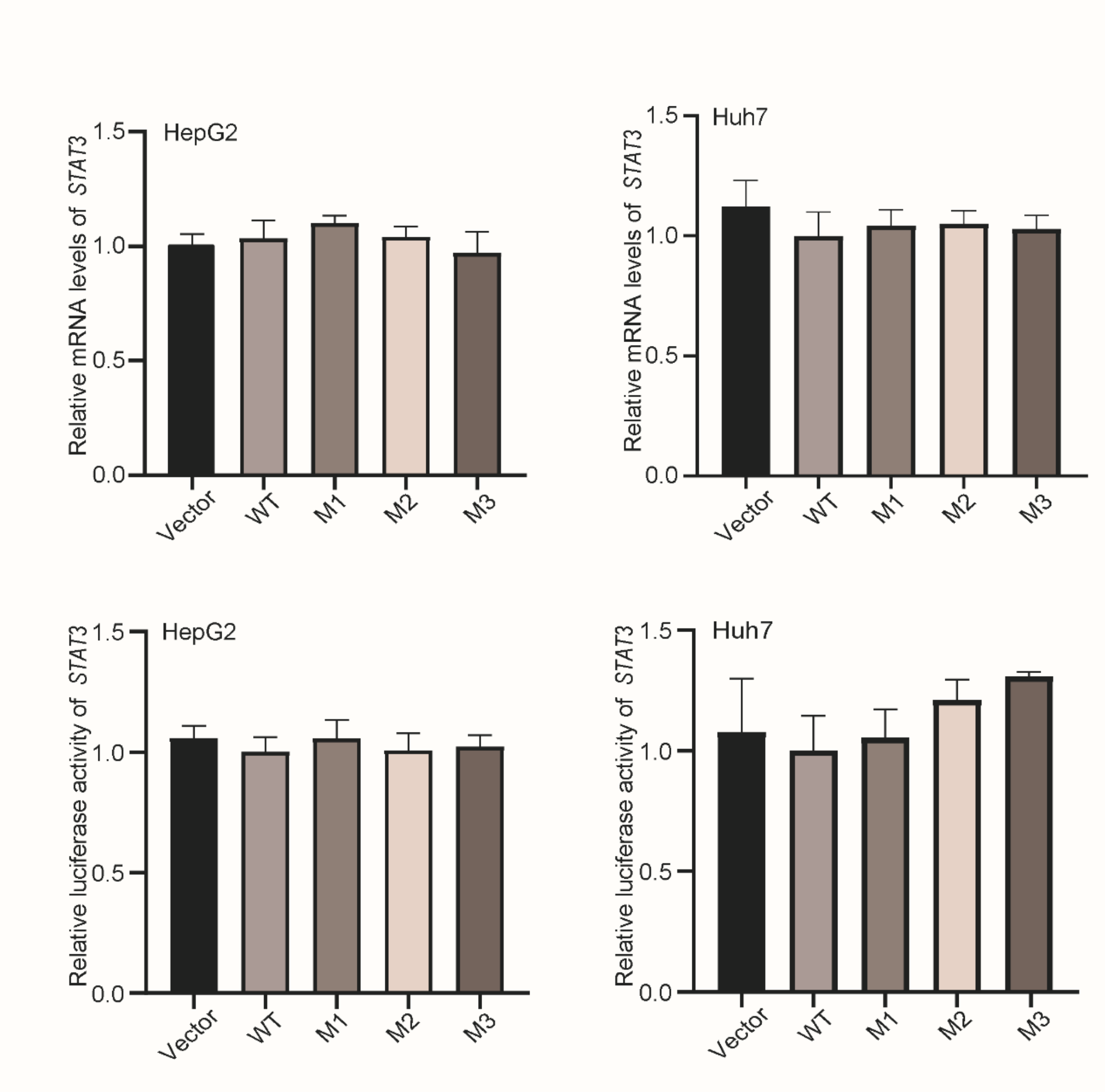

Figure S3. The expression levels of cytokines in mice serum. (A) The serum levels of TNFα, IL-1β, TGFβ1, and VEGF in SB mice with tumor (T) or those without tumor (N). (B) The serum levels of IL-4, IFNα, and GM-CSF in SB mice injected with empty vector, wild-type preS1/preS2/S fragment, or preS1/preS2/S mutants.

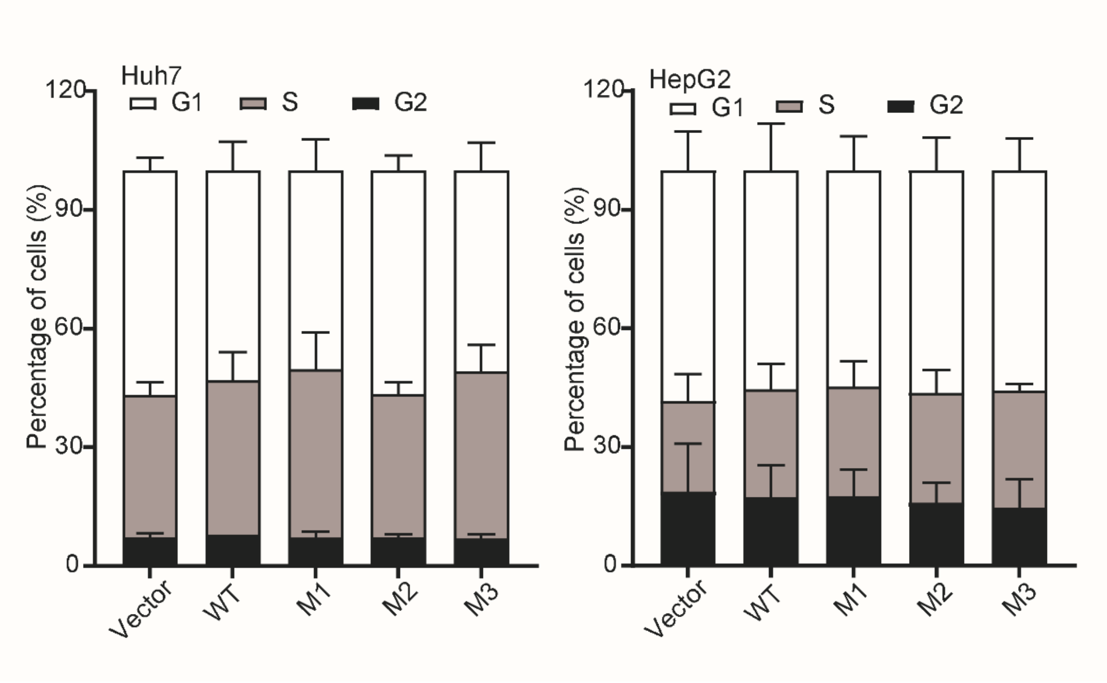

Figure S4. Results of cell cycle analysis. Ectopic expression of the preS1/preS2/S mutants had no significant effect on the cell cycle of Huh7 and HepG2 cells, compared to WT.

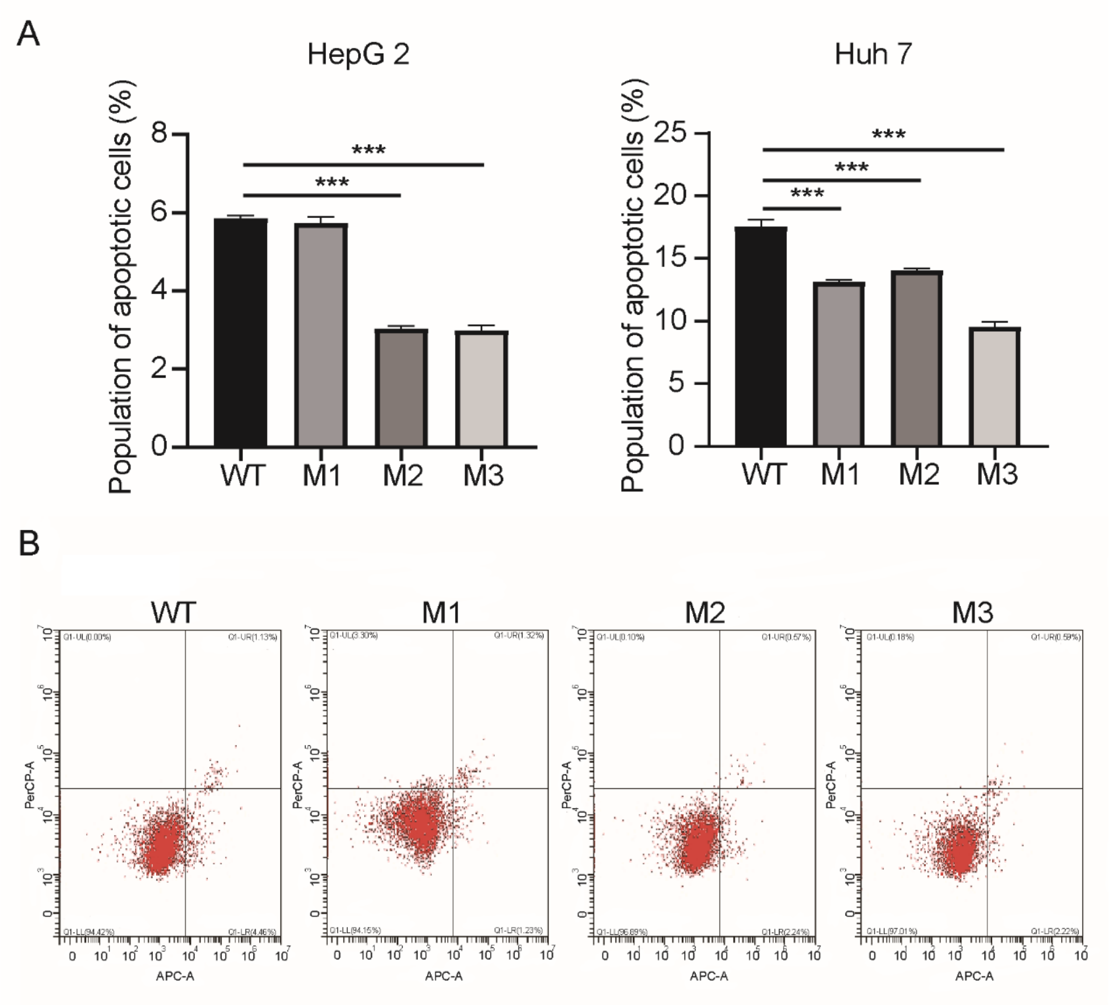

Figure S5. Apoptosis of HepG2 and Huh7 cells with ectopic expression of wild-type or mutant preS1/preS2/S. (A) Rates of total apoptotic cells are summarized in histogram plots. (B) Representative flow cytometry plots (HepG2 cells). Cells of total apoptosis were characterized as $APC^{+}/PerCP^{-}$ cells, $APC^{-}/PerCP^{+}$ cells, and $APC^{+}/PerCP^{+}$. n = 6, ***$P < 0.001$. Error bars represent standard deviation.

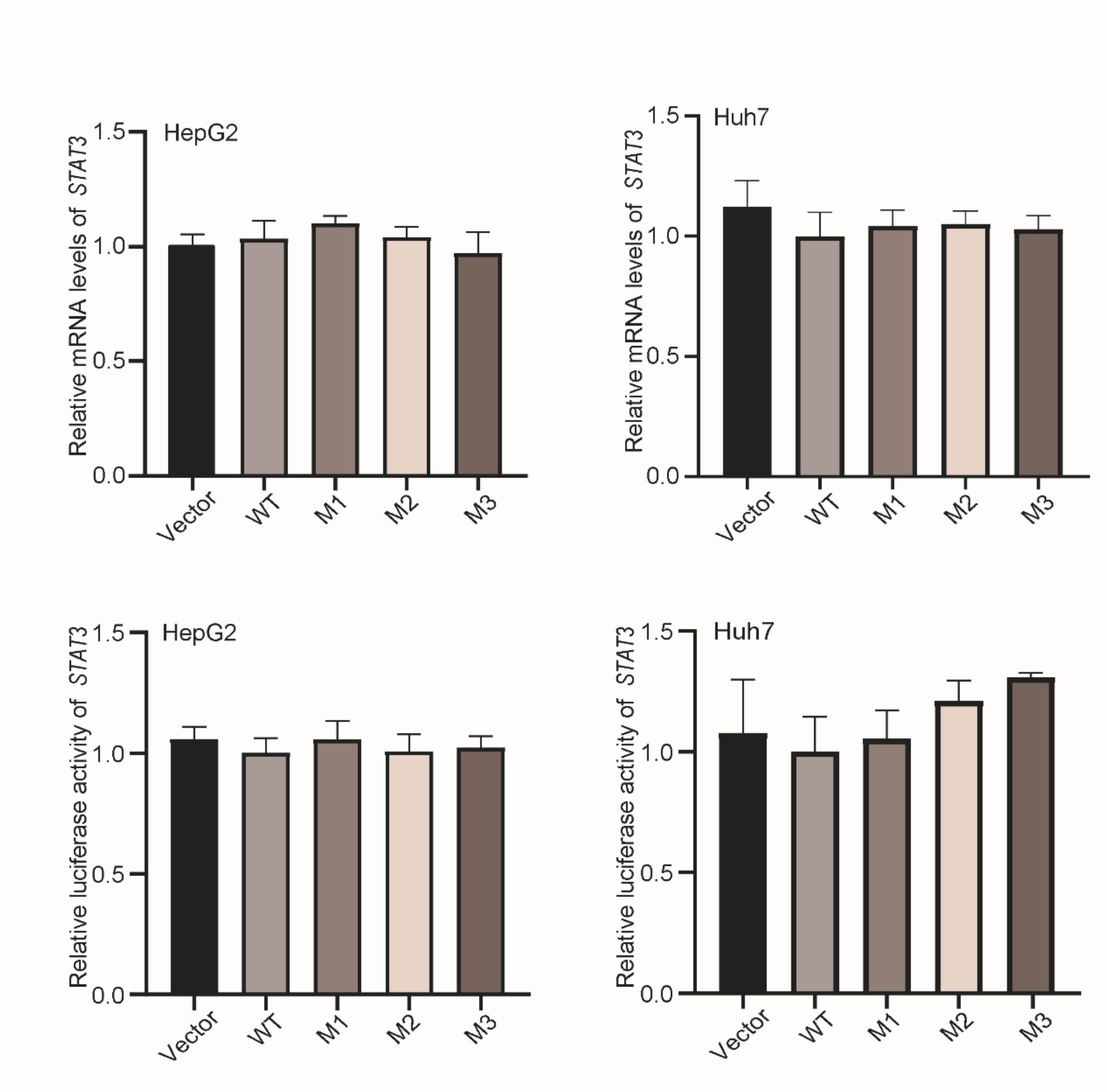

Figure S6. Effects of preS1/preS2/S mutants on the mRNA expression and promoter activity of STAT3. qRT-PCR demonstrated that the mRNA level of STAT3 was similar in cancer cells with ectopic expression of preS1/preS2/S variants. Luciferase assays demonstrated that the ectopic expression did not affect the promoter activity of STAT3 in Huh7 and HepG2 cells.

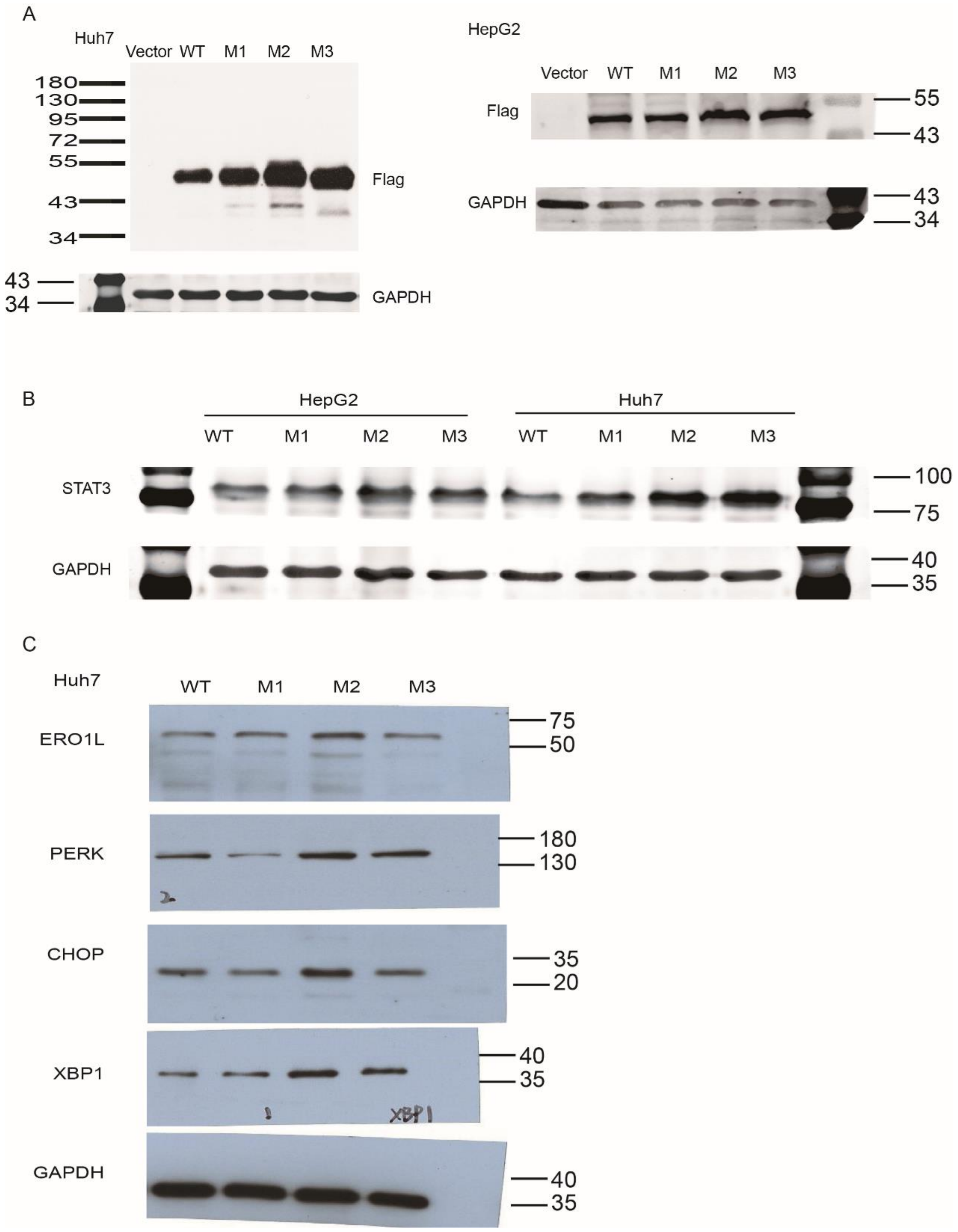

Figure S7. Original images for Western blot tests. (A) The original images corresponding to Figure 4A. (B) The original images corresponding to Figure 6A. (C) The original images corresponding to Figure 7A.

Table S1. Primers for the amplification and qRT-PCR.

Purpose	Sequence
preS nest PCR (first round)	Forward 5’-AAAATTAATTATGCCTGCTAGG-3’<br>Reverse 5’-GAGAAGTCCACCACGAGTC-3’
preS nest PCR (second round)	Forward 5’-TTTACAACTCTGTGGAAGGC-3’<br>Reverse 5’-GAGTCTAGACTCTGTGGTATTGG-3’
for construction of sleeping beauty plasmids	Forward 5’-CCGGAATTCCGG-ATGGGAGGTTGGTCTTCCAA-3’<br>Reverse 5’-CCGGAATTCCGG - TTAAATGTATACCCAAAGACA-3’
for *STAT3* qRT-PCR	Forward 5’-CAGCAGCTTGACACACGGTA-3’<br>Reverse 5’-TCTGTCTCACTAATTGCTCTCCT-3’
for *EHF* qRT-PCR	Forward 5’-CAGTGCAGTAGTGACCTGTTC-3’<br>Reverse 5’-CTGTGCTACCATAGTTGGTGTC-3’
for *PHGDH* qRT-PCR	Forward 5’-CTGCGGAAAGTGCTCATCAGT-3’<br>Reverse 5’-TGGCAGAGCGAACAATAAGGC-3’

for *WNT4* qRT-PCR	Forward 5’-AGGAGGAGACGTGCGAGAAA-3’<br>Reverse 5’-CGAGTCCATGACTTCCAGGT-3’
for *LAMC2* qRT-PCR	Forward 5’-GACAAACTGGTAATGGATTCCGC-3’<br>Reverse 5’-TTCTCTGTGCCGGTAAAAGCC-3’
for *MUC13* qRT-PCR	Forward 5’-CAGACAGTGAGTCAACCACAAA-3’<br>Reverse 5’-GGACCTGTGCTGTTTAGGGT-3’
for GAPDH qRT-PCR	Forward 5’-GGAGCGAGATCCCTCCAAAAT-3’<br>Reverse 5’-GGCTGTTGTCATACTTCTCATGG-3’

Table S2. preS mutations reported to be related with HCC risk

HBV Genotype	preS mutations
Only observed in genotype B	A76C, G49A, C52T, T105C, T109A, T147C, T2931C, A2950G, A2964C, G2962A, A2951G, T3026C, T3054A, G3063C, A3066T, G3069T, T3120A, G3186A, C3191G
Only observed in genotype C	A49G, T52C, C76A, C105T, A109T, C147T, C2931T, G2946A, G2950A, G2951A, A2962G, C2964A, C3026T, A3054T, C3063G, T3066A, T3069G, A3120T, A3120G, A3186G, G3191C
Observed in both genotype B and C	A7C, A10T, T31C, T53C, C135T, C3116T

Table S3. Antibodies for Western blot and Immunohistochemistry.

Proteins	Antibodies	Source	Catalog	Dilution ratio	References
CK-18 (for IHC)	Anti-Cytokeratin 18 antibody	Abcam	ab181597	1:800	Zhang S et al. Platelet-rich plasma improves therapeutic effects of menstrual blood-derived stromal cells in rat model of intrauterine adhesion. Stem Cell Res Ther 10:61 (2019).
STAT3 (for western blot and IHC)	Anti-STAT3 antibody	Abcam	ab68153	1:1000/1:200	Chen A et al. Inhibition of miR-155-5p attenuates the valvular damage induced by rheumatic heart disease. Int J Mol Med 45:429-440 (2020).
HBV S protein (for western blot and IHC)	Anti-HBS1L antibody	Abcam	ab224476	1:1000/1:200	This antibody has not yet been referenced specifically in any publications.
IL-6 (for western blot)	Anti-IL-6 antibody	Abcam	ab208113	1/50	Zhang J et al. Protocatechuic acid attenuates anterior cruciate ligament transection-induced osteoarthritis by suppressing osteoclastogenesis. Exp Ther Med 19:232-240 (2020).
Ki67 (for IHC)	Anti-Ki67 antibody	Abcam	ab16667	1/200	Traversi F et al. BRAFV600E Overrides NOTCH Signaling in Thyroid Cancer. Thyroid 31:787-799 (2021).

ERO1L<br>(for western blot and IHC)	Anti-ERO1L antibody	Abcam	ab177156	1:1000/1:200	Wang D et al. Tumor mutation burden as a biomarker in resected gastric cancer via its association with immune infiltration and hypoxia. Gastric Cancer 24:823-834 (2021).
PERK<br>(for western blot and IHC)	Anti-PERK antibody	Abcam	ab79483	1:1000/1:100	Sen T et al. Aberrant ER Stress Induced Neuronal-IFNß Elicits White Matter Injury Due to Microglial Activation and T-Cell Infiltration after TBI. J Neurosci 40:424-446 (2020).
XBP1<br>(for western blot)	Anti-XBP1 antibody	Abcam	ab37152	1:1000	Xu X et al. Endoplasmic reticulum stress/XBP1 promotes airway mucin secretion under the influence of neutrophil elastase. Int J Mol Med 47:81 (2021).
CHOP(for western blot)	Anti-CHOP antibody	CST	#2895	1:1000	Torres, Sandra et al. "Acid ceramidase improves mitochondrial function and oxidative stress in Niemann-Pick type C disease by repressing STARD1 expression and mitochondrial cholesterol accumulation." Redox biology vol. 45: 102052 (2021).
GAPDH<br>(For western blot)	Anti-GAPDH antibody	Abcam	ab8245	1:1000	Lin Q et al. Circular RNA ITCH downregulates GLUT1 and suppresses glucose uptake in melanoma to inhibit cancer cell proliferation. J Dermatolog Treat 32:231-235 (2021).

HBsAg (For Immunofluorescence staining)	Anti-HBsAg	Santa Cruz	sc-53299	1:800	Murphy CM et al. Hepatitis B Virus X Protein Promotes Degradation of SMC5/6 to Enhance HBV Replication. Cell Rep. 16:2846-2854 (2016).
Calnexin (For Immunofluorescence staining)	Anti-calnexin	Cell Signaling	# 2679	1:100	Shi L et al. Addiction to Golgi-resident PI4P synthesis in chromosome 1q21.3-amplified lung adenocarcinoma cells. Proc Natl Acad Sci U S A. 118:e2023537118 (2021).
Anti-mouse IgG (For Immunofluorescence staining)	Alexa488-conjugated donkey anti-mouse IgG	Invitrogen	A32766	1:1000	-
Anti-rabbit IgG (For Immunofluorescence staining)	Goat anti-Rabbit IgG, Cyanine3	Invitrogen	A10520	1:100	-
FLAG (for western blot)	Anti-FLAG antibody	Sigma-Aldrich	F1804	1:1000	

Table S4. Baseline characteristics and follow-up information of participants

Characteristics	Total (n=2114)	HBV genotype		Antiviral treatment		P value
		Genotype B (n=447)	Genotype C (n=1212)	No (n=1502)	Yes (n=612)	
Age (years)						
<60	1885(89.17)	412(92.17)	1082(89.27)	1308(87.08)	577(94.28)	
≥60	229(10.83)	35(7.83)	130(10.73)	194(12.92)	35(5.72)	0.924[a], 0.058[b], <0.001[c]
Gender						
Female	490(23.18)	89(19.91)	286(23.60)	383(25.50)	107(17.48)	
Male	1624(76.82)	358(80.09)	926(76.40)	1119(74.50)	505(82.52)	0.784[a], 0.110[b], 0.002[c]
Antiviral treatment						
No	1502(71.05)	289(64.65)	829(68.40)	-	-	
Yes	612(28.95)	158(35.35)	383(31.60)	-	-	0.108[a]
HBV genotype						
B	447(21.14)	-	-	289(19.24)	158(25.82)	
C	1212(57.73)	-	-	829(55.19)	383(62.58)	0.522[b], 0.313[c]
HBeAg positivity						
negative	1300(61.49)	269(60.18)	648(53.47)	1012(67.38)	288(47.06)	
positivity	814(38.51)	178(39.82)	564(46.53)	490(32.62)	324(52.94)	<0.001[a], <0.001[b], <0.001[c]
Cigarette smoking						

No	471(22.28)	116(25.95)	245(20.21)	342(22.77)	129(21.08)	
Yes	1643(77.72)	331(74.05)	967(79.79)	1160(77.23)	483(78.92)	0.163[a], 0.728[b], 0.528[c]
Alcohol drinking						
No	1699(80.37)	353(78.97)	987(81.44)	1193(79.43)	506(82.68)	
Yes	415(19.63)	94(21.03)	225(18.56)	309(20.57)	106(17.32)	0.450[a], 0.486[b], 0.023[c]
Cirrhosis						
No	1624(76.82)	392(87.70)	870(71.78)	1140(75.90)	484(79.08)	
Yes	490(23.18)	55(12.30)	342(28.22)	362(24.10)	128(20.92)	0.001[a], 0.520[b], 0.023[c]
HBV DNA (log10 copies/mL)						
<6.0	362(17.12)	36(8.05)	124(10.23)	318(21.17)	44(7.19)	
≥6.0	963(45.55)	241(53.91)	674(55.61)	594(39.55)	369(60.29)	<0.001[a], 0.075[b], <0.001[c]
Total bilirubin (mmol/L)						
≤20	509(24.08)	91(20.36)	305(25.17)	329(21.70)	180(29.41)	
>20	1580(74.74)	353(78.97)	886(73.10)	1160(77.23)	420(68.63)	0.428[a], 0.112[b], 0.007[c]
Direct bilirubin (mmol/L)						
≤7	691(32.69)	125(27.96)	427(35.23)	452(30.09)	239(39.05)	
>7	1396(66.04)	318(71.14)	764(63.04)	1035(68.91)	361(58.99)	0.113[a], 0.085[b], 0.003[c]
Albumin (g/L)						
≥35	1335(63.15)	302(67.56)	730(60.23)	921(61.32)	414(67.65)	
<35	772(36.52)	145(32.44)	475(39.19)	575(38.28)	197(32.19)	0.114[a], 0.273[b], 0.042[c]
Alanine aminotransferase (U/L)						
≤40	475(22.47)	71(15.88)	272(22.44)	381(25.37)	94(15.36)	
>40	1609(76.11)	371(83.00)	917(75.66)	1103(73.44)	506(82.68)	0.956[a], 0.049[b], <0.001[c]
Aspartate aminotransferase (U/L)						
≤37	380(17.98)	61(13.65)	198(16.34)	300(19.97)	80(13.07)	
>37	1514(71.62)	345(77.18)	875(72.19)	1010(67.24)	504(82.35)	0.283[a], 0.056[b], <0.001[c]

Platelet count (109/L)						
100-300	1004(47.49)	237(53.02)	517(42.66)	698(46.47)	306(50.00)	
<100	1110(52.51)	210(46.98)	695(57.34)	804(53.53)	306(50.00)	0.007[a], 0.010[b], 0.274[c]
Person-years of follow-up	23845	4977	13887	17361	6484	
Follow-up duration (years)a	11.67 (7.58-15.17)	11.50 (7.50-14.83)	11.83 (7.83-15.40)	12.33 (7.75-15.83)	10.67 (7.00-13.73)	
Hepatocellular carcinoma	224	27	137	184	40	
Incidence (/1000 person-years)b	9.39	5.42	9.87	10.60	6.17	

a Between Total and HBV genotype C;

b Between Total and Unantiviral treatment;

c Between Total and Antiviral treatment;

Table S5. Univariate and multivariate Cox regression analysis of factors significantly affected the occurrence of hepatocellular carcinoma in patients with genotype C HBV.

Variable	No. (%) of participants (n=1212)	Person-years of follow-up	No. of HCC (n=137)	Incidence rate per 1000 person-years	Univariate analysis HR (95% CI)	*P* Value	Multivariate analysis HR (95% CI)	*P* Value
Gender								
Female	286(23.60)	3591	17	4.73	1		1	
Male	926(76.40)	10296	120	11.66	2.37(1.43-3.95)	0.001	3.03(1.60-5.73)	0.001
Age (years)								
<60	1082(89.27)	12435	120	9.65	1		1	
≥60	130(10.73)	1452	17	11.71	1.26(0.76-2.10)	0.368	1.85 (0.92-2.46)	0.371
Antiviral therapy								
No	829(68.40)	9747	108	11.08	1		1	
Yes	383(31.60)	4140	29	7.00	0.60(0.40-0.90)	0.014	0.70(0.44-1.12)	0.139
Cirrhosis								
No	870(71.78)	10569	70	6.62	1		1	
Yes	342(28.22)	3318	67	20.19	3.13(2.24-4.38)	<0.001	2.21(1.35-3.62)	0.002
G2950A								
G	938(95.13)	10708	100	9.34	1		1	
A	48(4.87)	555	10	18.02	2.04(1.07-3.91)	0.032	1.23(0.75-2.01)	0.846
G2951A								
G	959(97.16)	10974	103	9.39	1			
A	28(2.84)	301	7	23.26	2.63(1.22-5.66)	0.013	0.98(0.10-9.42)	0.982

A2962G									
	A	961(97.07)	10992	101	9.19	1		1	
	G	29(2.93)	301	8	26.58	3.20(1.555-6.568)	0002	17.99(4.25-76.12)	0.040
C2964A									
	C	965(97.28)	11005	104	9.45	1		1	
	A	27(2.72)	292	6	20.55	2.37(1.04-5.39)	0.004	0.19(0.01-2.54)	0.211
Direct bilirubin (mol/L)									
	≤7	427(35.85)	5191	35	6.74	1		1	
	>7	764(64.15)	8458	101	11.94	1.8(1.23-2.64)	0.003	0.97(0.61-1.53)	0.882
Albumin (g/L)									
	≥35	730(60.58)	9029	62	6.87	1			
	<35	475(39.42)	4775	73	15.29	2.26(1.61-3.17)	<0.001	1.24(0.76-2.00)	0.388
α-fetoprotein (ng/mL)									
	≤20	731(60.46)	8694	69	7.94	1		1	
	20-400	478(39.54)	5154	68	13.19	1.64(1.18-2.30)	0.004	1.25(0.83-1.87)	0.283
Platelet count (10^9/L)									
	100-300	517(42.66)	6361	37	5.82	1		1	
	<100	695(57.34)	7526	100	13.29	2.32(1.59-3.38)	<0.001	1.23(0.75-2.01)	0.410

Table S6. Frequently integrated genes of the sleeping beauty mice models

Gene	Frequency of integration	Functional region	Integration depth†	Sample-ID
Mast4	2/6	intron	0.0029, 0.0023	M2-cancer-1<br>M2-cancer-2
Tmem178b	2/6	intron	0.024, 0.00085	M2-cancer-1<br>M2-cancer-2
Zbtb20	2/6	intron	0.00079, 0.0006	M2-cancer-1<br>M2-cancer-2

† ratio of integrated reads to normal reads

Table S7. qRT-PCR validation of 5 differential expressed genes specifically identified in the cDNA microarray data of SB mice

Gene	Results of cDNA microarray analysis in *SB* mice Average fold change (*P* value)	Results of RT-qPCR validation Average fold change (*P* value)		
		IL-5-stimulated Huh7 cells vs control Huh7 cells	IL-6-stimulated Huh7 cells vs control Huh7 cells	preS1/preS2/S mutants-expressing Huh7 cells vs WT-preS1/preS2/S- expressing Huh7 cells
WNT4	12.66 (0.004)[1], 5.67 (0.001)[2], 2.59 (0.003)[3]	2.13 (<0.001)	2.95 (<0.001)	1.06 (0.99)[1],0.92 (0.17)[2],1.10 (0.99)[3]
EHF	3.50 (<0.001)[1], 2.88 (0.003)[2], 5.99 (<0.001)[3]	2.30 (<0.001)		1.32 (0.27)[1], 1.06 (0.99)[2],1.18 (0.99)[3]
PHGDH	2.76 (0.002)[1], 3.22 (0.009)[2], 2.38 (0.004)[3]	1.51 (<0.001)		0.84 (0.72)[1], 1.26 (0.22)[2], 1.11 (0.99)[3]
LAMC2	2.20 (0.020)[1], 4.86 (0.045)[2], 3.11 (0.005)[3]		4.53 (<0.001)	0.99 (0.99)[1], 1.03 (0.99)[2], 1.16 (0.51)[3]
MUC13	6.60 (0.004)[1], 2.81 (0.035)[2], 2.69 (0.008)[3]		1.89 (0.01)	1.24 (0.09)[1], 1.07 (0.99)[2], 1.18 (0.26)[3]

[1] M1 vs WT; [2] M2 vs WT; [3] M3 vs WT;

References:

1. Chen J, Yin J, Tan X, Zhang H, Zhang H, Chen B, et al. Improved multiplex-PCR to identify hepatitis B virus genotypes A-F and subgenotypes B1, B2, C1 and C2. J Clin Virol. 2007;38:238-43.
2. Jianhua Yin, Jiaxin Xie, Shijian Liu, Hongwei Zhang, Lei Han, Wenying Lu, et al. Association between the various mutations in viral core promoter region to different stages of hepatitis B, ranging of asymptomatic carrier state to hepatocellular carcinoma. Am J Gastroenterol. 2011;106(1):81-92.
3. Li X, Tan X, Yu Y, Chen H, Chang W, Hou J, et al. D9S168 microsatellite alteration predicts a poor prognosis in patients with clear cell renal cell carcinoma and correlates with the down-regulation of protein tyrosine phosphatase receptor delta. Cancer. 2011;117:4201-4211.
4. Chang W, Gao X, Han Y, Du Y, Liu Q, Wang L, et al. Gene expression profiling-derived immunohistochemistry signature with high prognostic value in colorectal carcinoma. Gut 2014;63:1457-1467.
5. Wang Q, Jia P, Zhao Z. VERSE: a novel approach to detect virus integration in host genomes through reference genome customization. Genome Med 2015;7:2.
6. Wang K, Li M, Hakonarson H. ANNOVAR: functional annotation of genetic variants from high-throughput sequencing data. Nucleic Acids Res 2010;38:e164.
7. McCarthy DJ, Chen Y, Smyth GK. Differential expression analysis of multifactor RNA-Seq experiments with respect to biological variation. Nucleic Acids Res 2012;40:4288-4297.
8. Yang D, Li Q, Shang R, Yao L, Wu L, Zhang M, et al. WNT4 secreted by tumor tissues promotes tumor progression in colorectal cancer by activation of the Wnt/β-catenin signalling pathway. J Exp Clin Cancer Res. 2020;39(1):251.
9. Gu ML, Zhou XX, Ren MT, Shi KD, Yu MS, Jiao WR, et al. Blockage of ETS homologous factor inhibits the proliferation and invasion of gastric cancer cells through the c-Met pathway. World J Gastroenterol. 2020;26(47):7497-7512.
10. Chandrika M, Chua PJ, Muniasamy U, Huang RYJ, Thike AA, Ng CT, et al. Prognostic significance of phosphoglycerate dehydrogenase in breast cancer. Breast Cancer Res Treat. 2021;186(3):655-665.
11. Ang H, Cai J, Du S, Wei W, Shen X. LAMC2 modulates the acidity of microenvironments to promote invasion and migration of pancreatic cancer cells via regulating AKT-dependent NHE1 activity. Exp Cell Res. 2020;391(1):111984.
12. Tiemin P, Fanzheng M, Peng X, Jihua H, Ruipeng S, Yaliang L, et al. MUC13 promotes intrahepatic cholangiocarcinoma progression via EGFR/PI3K/AKT pathways. J Hepatol. 2020;72(4):761-773.
